# Dissection of the epoxyjanthitrem pathway in *Epichloë* sp. *Lp*TG-3 strain AR37 by CRISPR gene editing

**DOI:** 10.3389/ffunb.2022.944234

**Published:** 2022-08-10

**Authors:** Taryn A. Miller, Debbie A. Hudson, Richard D. Johnson, Jaspreet S. Singh, Wade J. Mace, Natasha T. Forester, Paul H. Maclean, Christine R. Voisey, Linda J. Johnson

**Affiliations:** Grasslands Research Centre, AgResearch, Palmerston North, New Zealand

**Keywords:** secondary metabolite, gene editing, endophyte, footprint-less, mass spectrometry, bioinformatics, off-targets, indole diterpene

## Abstract

*Epichloë festucae* var. *lolii* and *Epichloë* sp. *Lp*TG-3 are filamentous fungal endophytes of perennial ryegrass (*Lolium perenne*) that have a substantial impact on New Zealand’s agricultural economy by conferring biotic advantages to the host grass. Overall, *Epichloë* endophytes contribute NZ$200 million to the economy annually, with strain AR37 estimated to contribute NZ$3.6 billion to the New Zealand economy over a 20-year period. This strain produces secondary metabolites, including epoxyjanthitrems, which are a class of indole diterpenes, associated with the observed effects of AR37 on livestock and insect pests. Until very recently, AR37 was intractable to genetic modification but this has changed with the application of CRISPR-Cas9 based gene editing techniques. In this paper, gene inactivation by CRISPR-Cas9 was used to deconvolute the genetic basis for epoxyjanthitrem biosynthesis, including creating an AR37 strain that has been edited to remove the biosynthesis of all indole diterpenes. We show that gene editing of *Epichloë* can be achieved without off-target events or introduction of foreign DNA (footprint-less) through an AMA1-based plasmid that simultaneously expresses the CRISPR-Cas9 system and selectable marker. Genetic modification events in these transformants were investigated through genome sequencing and *in planta* chemistry.

## Introduction

*Epichloë* endophytes have been successfully commercialised as a seed-borne technology for pastoral agriculture to confer host persistence from invertebrate and vertebrate grazing pressures in New Zealand, and to a lesser extent, Australia and the USA (Johnson et al., 2013a). The symbiotic association that these filamentous fungi form with Pooideae grasses such as perennial ryegrass (*Lolium perenne*) are particularly crucial for conferring insect bioprotection in New Zealand farming systems (Caradus and Johnson, 2019; Johnson and Caradus, 2019). A widely adopted *Epichloë* strain for perennial ryegrass pastures in New Zealand is AR37 (*Lolium perenne* taxonomic group 3, *Lp*TG3). This endophyte strain provides protection against Argentine stem weevil (*Listronotus bonariensis*), pasture mealy bug (*Balanococcus poae*), porina (*Wiseana* spp), black beetle (*Heteronychus arator*), and root aphid (*Aploneura lentisci*). Of the known secondary metabolite pathways in *Epichloë*, AR37 has a non-functional peramine pathway and a functional indole diterpene (epoxyjanthitrems) pathway, with both the ergot alkaloid and loline pathways absent (Johnson et al., 2013a).

Indole diterpenes are a class of secondary metabolite compounds comprised of a cyclic diterpene-derived skeleton joined to an indole moiety that are produced by filamentous fungi from the phylum Ascomycota e.g., *Penicillium, Aspergillus, Claviceps*, and *Epichloë*. Structural and therefore bioactive diversity is brought about through differences in stereochemistry, prenylation, hydroxylation, epoxidation, methylation, and oxidation patterns (Kozák et al., 2019). All species that produce indole diterpenes contain a core set of four genes that code enzymes involved in the production of the first stable intermediate; IdtG (geranylgeranyl pyrophosphate synthase) converts isopentenyl diphosphate (IPP) and/or dimethylallyl pyrophosphate (DMAPP) and/or geranyl pyrophosphate (GPP) and/or farnesyl pyrophosphate (FPP) into geranylgeranyl pyrophosphate (GGPP). IdtC (a geranylgeranyl transferase) catalyses the indole condensation of GGPP with indole-3-glycerol phosphate (IGP) to produce 3-geranylgeranyl indole (3-GGI). IdtM (a FAD dependent epoxidase) either single or double epoxidates, depending on species, the 3-GGI, which is then finally cyclised by IdtB (a cyclase) *via* either a mechanism of Markovnikov’s or anti-Markovnikov’s rule, again species dependent. In *Epichloë*, all indole diterpene pathways are double epoxidated and cyclised through the Markovnikov’s mechanism to produce the first stable intermediate paspaline (Parker and Scott, 2004).

In *Epichloë*, the indole diterpene pathway produces two well characterised classes of decorated indole diterpenes, the lolitrems and the epoxyjanthitrems. These two classes of indole diterpenes are produced by a core set of genes as well as a small further subset of unique genes and are typically only expressed *in planta* (Young et al., 2009; Ludlow et al., 2019; Finch et al., 2020). The lolitrems are produced in a range of *Epichloë* species, with diversification through non-functional genes or the presence or absence of genes (Young et al., 2009), with lolitrem B responsible for ryegrass staggers, a neurological disorder of grazing livestock (Fletcher and Harvey, 1981). This pathway consists of an interconnected network of compounds, rather than a linear pathway, synthesised by 11 genes in total grouped into 3 clusters interspaced with AT-rich regions in a single sub-telomeric loci (Young et al., 2006; Saikia et al., 2012). Further investigation has been conducted into identifying the exact pathway intermediates between paspaline and lolitrem B, including structure, pathway placement, tremorgenicity, and insect bioactivity, *e.g.* lolitrems A, B, and F have long-term tremors while lolitrem E is non-tremorgenic, to build up an understanding of this important bioactive secondary metabolite pathway (Saikia et al., 2008; Saikia et al., 2012). In contrast, epoxyjanthitrems are produced in a restricted group of strains within the taxonomic grouping *Lp*TG-3 (Ludlow et al., 2019) (Linda Johnson, unpublished data). These compounds are associated with mild ryegrass staggers but more importantly bioactivity against porina (Hennessy et al., 2016; Finch et al., 2020). Recently, a single loci consisting of 16 genes associated into 4 clusters was identified as potentially being involved in epoxyjanthitrem biosynthesis through bioinformatic analysis of the genome of *Epichloë* sp. *Lp*TG-3 strain NEA12 (Ludlow et al., 2019). Clusters 1 and 2 were analogous to the first two clusters in the lolitrem pathway (Young et al., 2009), cluster 3 was novel and linked to epoxyjanthitrem biosynthesis through RNAi knock-down of *jtmD (idtD)* followed by *in planta* chemistry, and cluster 4 was also novel but no additional experimental evidence was collected. A network pathway was then proposed based on the *jtmD* RNAi chemistry with proposed gene functions, and indole diterpene structures from this study (Ludlow et al., 2019). In addition, the chemical structures of all five epoxyjanthitrem compounds have now been elucidated (Finch et al., 2020).

Traditionally, genetic modification of *Epichloë* has involved the use of homologous recombination in conjunction with protoplast mediated transformation to either knock-out or knock-in genes. Sexual species such as the model *E. festucae* strain Fl1, are genetically tractable to these techniques allowing a thorough investigation into the genes involved in secondary metabolite biosynthesis as well as the *Epichloë*-ryegrass symbiotic association (Fleetwood et al., 2007; Eaton et al., 2008; Tanaka et al., 2008; Charlton et al., 2012; Saikia et al., 2012; Johnson et al., 2013b; Tanaka et al., 2013; Berry et al., 2015; Johnson et al., 2015; Voisey et al., 2016; Rahnama et al., 2018; Scott et al., 2018; Hassing et al., 2019; Rahnama et al., 2019; Lukito et al., 2020; Noorifar et al., 2021; Johnson et al., 2021; Hassing et al., 2022). However, in general, these techniques have been less effective on asexual strains such as AR37 and AR1, making molecular investigation into these strains near impossible (Panaccione et al., 2001) (Linda Johnson, unpublished).

The CRISPR (Clustered Regularly Interspaced Short Palindromic Repeats)/Cas (CRISPR Associated protein) system is a naturally derived immune system originally discovered from bacteria and archaea, and is divided into two Classes, I and II, each further classified by sub-class I-VI. For example, Cas9 is a class II sub-class II CRISPR/Cas enzyme system (Jinek et al., 2012; Makarova et al., 2018). By 2013 CRISPR-Cas had been adapted as a novel gene editing technique in a range of systems *e.g.*, mammals, plants, insects, and fungi (Jiang et al., 2021). More recently, Song et al. (2018) developed a sophisticated CRISPR-Cas9 delivery system that has been successfully used in *Aspergillus via* an autonomously replicating plasmid ANEp8_Cas9_LIC that simultaneously expresses the Cas9 protein, associated guide, and selectable marker for CRISPR genetic modification (Kun et al., 2021). CRISPR has been successfully implemented in other *Epichloë* species through alternative expression systems (Florea et al., 2021; Wang et al., 2021). The CRISPR-Cas system used by Wang et al. (2021) was based on a single plasmid harbouring a chimeric RNA guide and the *cas9* gene under a fungal promoter, however without the autonomously replicating AMA sequence present in the vector delivery system used by Song et al. (2018). In contrast the approach taken by Florea et al. (2021) was based on Cas9-sgRNA (single guide and tracrRNA) ribonucleoprotein complexes (RNPs).

In this paper, we used a modification of the CRISPR-Cas9 autonomously replicating plasmid, developed by Song et al. (2018), to inactivate genes in the epoxyjanthitrem pathway of AR37 in order to i) investigate a novel method of genetic modification of AR37, ii) investigate the footprint-less and off-target nature of this specific CRISPR-Cas9 system, iii) compare the epoxyjanthitrem genes in AR37 with those in NEA12 (Ludlow et al., 2019) through bioinformatic analysis of the genome, iv) confirm involvement of *ltmM* (hence forth *idtM)*, *jtmO* (hence forth *idtO*), *jtm02* (hence forth *idtA*), and *ltmF* (hence forth *idtF*) in epoxyjanthitrem synthesis, and v) provide further validation of the proposed epoxyjanthitrem pathway, which we have determined differs to the previously published version by Ludlow (2019).

## Methods

### Bacterial strains and growth conditions

*Escherichia coli* strains Top10 and One Shot™ *ccd*B survival™ 2 T1^R^ competent cells (Invitrogen Corp., Carlsbad, California, USA) were grown at 37°C at 180 rpm in Luria–Bertani broths or 1.5% (*w*/*v*) agar plates (made to manufactures specifications) supplemented where necessary with ampicillin (100 µg/mL).

### Fungal strains and growth conditions

Cultures of *Epichloë* were maintained on 1.5% (*w*/*v*) potato dextrose agar (PDA) plates (Difco, Sparks, Maryland, USA) (made to manufactures specifications) supplemented where necessary with hygromycin (150 µg/mL) and grown at 22°C at 180 rpm.

### Adaptions to the ANEp8_Cas9_LIC1 plasmid to create Cas9HygAMAccdB

The ANEp8_Cas9_LIC plasmid (Song et al., 2018) (**Figure S1A** and **Table S1**) was constructed through the introduction of a 38-bp ligation independent cloning (LIC) site, centred with a *Swa*I restriction site, into the ANEp8_Cas9 plasmid described in Song et al. The ANEp8_Cas9_gRNA plasmid (obtained from Concordia University) (**Figure S1B** and **Table S1**) was constructed through the introduction of a gRNA cassette, *via* the LIC method, into ANEp8_Cas9_LIC shown in Song et al. - **Figure S5** We adapted the ANEp8_Cas9_LIC plasmid to contain a hygromycin cassette in place of the *PyrG* gene for selection. The ANEp8_Cas9_LIC plasmid was initially digested with *NotI* to liberate a 5.3 kb fragment containing the AMA1 cassette (purified by gel extraction) and a 10.3 kb fragment (purified by gel extraction) containing the *Cas9* and *PyrG* genes. Subsequent digestion of the 10.3 kb fragment with *KpnI* liberated a 9 kb Cas9 cassette (purified by gel extraction) containing the *Cas9* gene and removal of *PyrG*. To amplify the hygromycin resistance cassette (2.9 kb), primer PCR was performed on pDONR221-Hyg template with restriction enzyme adapted primers *Kpn*I TtrpC Hyg DONR R and *Not*I PgpdA Hyg pDONR F (**Table S1**), with the resulting product being digested with *KpnI* and *NotI* prior to ligation. The Cas9 (*NotI/KpnI* digested) cassette and the Hygromycin resistance (*NotI/KpnI* digested) cassette were ligated with T4 ligase (Invitrogen Corp., Carlsbad, California, USA) at 16°C overnight, creating the Cas9Hyg plasmid. The Cas9Hyg plasmid was re-digested with *NotI* to linearise and alkaline phosphatase treated (purified by gel extraction) before its T4 ligation with the AMA1 (*NotI* digested) cassette, creating the Cas9HygAMA plasmid. The addition of the gRNA cassette containing the mock sgRNA *SapI* protospacer was achieved through fusion PCR and LIC. The plasmid ANEp8-Cas9-gRNA was used as a template in the gRNA cassette construction which was assembled by fusion PCR of the two DNA fragments using the primers: *Sap*I site CRISPR Fw P1 and Rev LIC2 as well as *Sap*I site CRISPR Rev P1 and Fw LIC2 (**Table S1**). The first fragment containing the tRNA promoter was amplified from ANEp8-Cas9-gRNA plasmid and the 20 bp protospacer sequence containing the *Sap*I sites was added at the 3’-end through PCR using an overlapping sequence (**Table S1**). The second fragment was amplified from ANEp8-Cas9-gRNA plasmid with the 20 bp *Sap*I protospacer sequence and tRNA terminator sequence added at 5’and 3’ends respectively *via* PCR (**Table S1**). In the fusion, PCR 1ng of each of the gel extracted PCR fragments were used as template in a 25 μL reaction and performed with Phusion DNA polymerase (New England Biolabs (NEB, Ipswich, Massachusetts, USA). The resulting PCR product was inserted into the Cas9HygAMA plasmid through LIC *via* its *SwaI* restriction enzyme site creating Cas9HygAMASapI. A *ccdB* lethal cassette was cloned between the two *SapI* sites to aid in the efficiency of future protospacer cloning. To amplify the *ccdB* lethal cassette sequence (2 kb), primer PCR was performed on the split marker plasmid pDONR-SM1 template with *Sap*I restriction enzyme adapted primers *Sap*I ccdB F and *Sap*I ccdB R (**Table S1**). The resulting product was digested with *SapI* prior to its ligation with Cas9HygAMASapI (*Sap*I digested) creating the Cas9HygAMAccdB (**Figure S1C** and **Table S1**) plasmid.

### CRISPR-Cas9 protospacer design and cloning

The AR37 epoxyjanthitrem genes *idtM*, *idtD, idtO, idtA, and idtF* were screened for CRISPR-Cas9 target sites N(21)GG, with the 3′ PAM sequence (NGG) using Geneious (2019.1.1) (Biomatters Ltd, Auckland, New Zealand). Two protospacer sequences targeting each gene (with no predicted off target-sites) were selected (**Table 1**) (Doench et al., 2014; Xie et al., 2014).

**Table 1:** CRISPR guide parameters.

AR37 strain		Geneious software				Transformation		Chemistry		
Gene	Guide	PAM	Doench score (Doench et al., 2014)	Zhang score (Xie et al., 2014)	Off-targets	Inactivation rate		Transformant	CRISPR indel	Frameshift
*idtM*	g32	CGG	0.791	100%	0 (0 in CDS)	18%	(3/16)	#12	C insertion	1 bp
*idtM*	g38	CGG	0.696	100%	0 (0 in CDS)	68%	(11/16)	#8	A deletion	7 bp
*idtD*	g63	TGG	0.748	100%	0 (0 in CDS)	62%	(5/8)	#113	T insertion	2 bp
*idtD*	g148	CGG	0.735	100%	0 (0 in CDS)	100%	(1/1)	#9	A insertion	33 bp
*idtO*	g119	CGG	0.652	100%	0 (0 in CDS)	10%	(1/10)	#1	A insertion	12 bp
*idtO*	g144	CGG	0.812	100%	0 (0 in CDS)	100%	(7/7)	#8	A insertion	57 bp
*idtA*	g101	CGG	0.712	100%	0 (0 in CDS)	33%	(3/9)	#3-4	AA deletion	147 bp
*idtA*	g64	GGG	0.779	100%	0 (0 in CDS)	15%	(3/20)	#19-9	T insertion	20 bp
*idtF*	g86	TGG	0.714	100%	0 (0 in CDS)	0%	(0/3)	–	–	–
*idtF*	g119	GGG	0.767	100%	0 (0 in CDS)	75%	(3/4)	#4	C deletion	20 bp
*idtA/idtF*	g64/g119	–	–	–	–	75%	(3/4)	#4	C insertion	0 bp
*idtA/idtF*	g101/g86	–	–	–	–	33%	(3/9)	#8	G deletion	10 bp

### Construction of plasmids Cas9HygAMA-idtMg32, Cas9HygAMA-idtMg38, Cas9HygAMA-idtDg63 and Cas9HygAMA idtDg148

The ANEp8-Cas9-LIC (**Figure S1A** and **Table S1**) plasmid was used as host plasmid to harbour the gRNA cassette by using the LIC method (Song et al., 2018). The *idtM* and *idtD* gRNA cassette used for plasmid construction was amplified with a pair of end primers to link with LIC sequence sites at both sides (*idtM* g32 Rev P1/*idtM* g32 Fw P1 = g32, *idtM* g38 Rev P1/*idtM* g38 Fw P1 = g38, *idtD* g63 Rev P1/*idtD* g63 Fw P1 = g63, and *idtD* g148 Rev P1/*idtD* g148 Fw P1 = g148) (**Table S1**) to generate complementary single-strand overhangs between ANEp8-Cas9 plasmid and the *idtM*/*idtD* gRNA cassette insert. The *Swa*I linearised ANEp8-Cas9 and the *idtM/idtD* gRNA cassette DNA (ending with LIC tails) were treated by T4 DNA polymerase in the presence of dGTP and dCTP, respectively. The 20 μL reaction mixture contained 0.2 pmol of DNA, 0.8 μL of 100 mM dithiothreitol, 2 μL of 25 mM dGTP or dCTP, and 3 U of T4 DNA polymerase in NEB buffer 2.1. The reaction was carried out at 22°C for 30 mins followed by enzyme inactivation by heating at 75°C for 20 mins. The insert and plasmid were mixed in a 3:1 molar ratio. To achieve annealing, the mixture was first heated at 60°C for 5 mins and then gradually decreased to 4°C (reduce 0.1°C per second). The annealed products were transformed into *E*.*coli* Top10 (Invitrogen Corp., Carlsbad, California, USA) competent cells to generate plasmids Cas9HygAMA-*idtM*g32 (**Figure S1D**), Cas9HygAMA-*idtM*g38, Cas9HygAMA-*idtD*g63, and Cas9HygAMA-*idtD*g148 (**Table S1**). Transformants were pre-screened by PCR using PrimeSTAR GXL polymerase (Takara Bio Inc, USA) with the gRNA screen F and gRNA screen R primers (**Table S1**). ZymoPure large plasmid prep kit (Zymo Research, Orange, California, USA) was used to obtain the plasmids and these were checked by sequencing using the gRNA screen F and gRNA screen R primers (**Table S1**).

### Construction of plasmids Cas9HygAMA-idtOg119, Cas9HygAMA-idtOg144, ANEp8-Cas9-idtAg64, ANEp8-Cas9-idtAg101, ANEp8-Cas9-idtFg86, and ANEp8-Cas9-idtFg119

Primers were designed to contain 20 bp guide protospacer sequences for the *idtO* (idtO g119 Top/idtO g119 Btm = g119 and idtO g144 Top/idtO g144 Btm = g144), *idtA* (idtA g101 Top/idtA g101 Btm = g101 and idtA g64 Top/idtA g64 Btm = g64), and *idtF* (idtF g86 Top/idtF g86 Btm = g86 and idtF g119 Top/idtF g119 Btm = g11) (**Table S1**) genes, with the appropriate *Sap*I overhang at each end for cloning into the Cas9HygAMAccdB (**Figure S1C**) plasmid. Each protospacer was generated by annealing 15ng of the forward and reverse oligonucleotides in annealing buffer (10 mM Tris-HCl pH 8, 50 mM NaCl, 1 mM EDTA, pH 8). The following thermocycler program was used for annealing: 5 mins at 95°C, 20 sec at 95°C, a decrease of 0.5°C/cycle for 140 cycles, 1 mins at 25°C. The annealed oligonucleotides were ligated with the digested Cas9HygAMAccdB (*Sap*I) plasmid using T4 ligase (Invitrogen Corp., Carlsbad, California, USA) at 20°C for 15 mins. The annealed products were transformed into *E.coli* Top10 competent cells to generate Cas9HygAMA-idtOg119 and Cas9HygAMA-idtOg144 for the *idtO* gene, Cas9HygAMA-idtAg64 and Cas9HygAMA-idtAg101 for the *idtA* gene, and Cas9HygAMA-idtFg86 and Cas9HygAMA-idtFg119 for the *idtF* gene (**Table S1**). Transformants were screened by colony PCR using Sapphire Amp Fast PCR master mix (Takara Bio Inc, USA) with the *idtO/idtA/idtF* gRNA Top anneal oligonucleotide and the reverse gRNA-specific primer gRNA Screen R (**Table S1**). Sequencing to confirm the correct gRNAs was achieved by primer PCR using the gRNA screen F and gRNA screen R primers (**Table S1**).

### Genomic DNA/plasmid isolation and sequencing

Genomic DNA for PCR screening was isolated from *Epichloë* mycelium using the Fungal Bacterial DNA mini kit (Zymo Research, Orange, California, USA). Genomic DNA was extracted from AR37 *idtM*, *idtD*, and *idtA* CRISPR gene edited strains using modified small scale Byrd et al. (1990) method: where the chloroform:phenol extraction steps were replaced by the Fungal Bacterial DNA mini kit (Zymo Research, Orange, California, USA). Plasmid DNA was isolated and purified from *E. coli* cultures using the ZymoPure large Plasmid Prep kit (Zymo Research, Orange, California, USA). Plasmids and transformants were sequenced at Massey Genome Service (Palmerston North, New Zealand) using the ABI 3730 and the ABI 3500xl DNA Analyser, with data analysed using Geneious (2019.1.1) software. The genomic DNA was sequenced at Massey Genome Service (Palmerston North, New Zealand) by single lane of 250 bp paired-end sequence on an Illumina MiSeq. The reads were dynamically trimmed using the SolexaQA package to their longest fragment such that the base call error rates did not exceed a P value of 0.05, and paired end reads of less than 100 bp discarded.

### Fungal protoplast isolation and transformation

Protoplasts of *Epichloë* were prepared as described in Fleetwood et al. (2007). AR37 wild type protoplasts were transformed as described in Fleetwood et al. (2007) with 300 fmol of the appropriate Cas9HygAMAgRNA plasmid, or for *idtA/idtF* inactivated gene edits, AR37 inactivated *idtA* protoplasts were transformed with the corresponding *idtF* Cas9HygAMAgRNA plasmid (**Table S1**). Transformants were selected on RG Media (PD with 0.8 M sucrose pH 6.5) containing hygromycin (150 µg/mL). To obtain clonal isolates, the resulting transformants were purified by sub-culturing two times as described by Young et al. (2005) and screened by PCR for CRISPR gene edits as detailed in **Table S1**, with gene editing events confirmed by sequencing.

### Endophyte inoculation

For each guide, a single transformant predicted to result in early translational termination due to a frame shift in the predicted functional domain (*i.e.*, non-functional transcript) was selected for inoculation in preparation for *in planta* chemistry. Endophyte-free seedlings were inoculated using the method of Latch and Christensen (1985) as follows: Perennial ryegrass (*Lolium perenne* cv. Samson) were inoculated with *Epichloë* AR37 wild type or CRISPR-Cas9 gene edited strains. Seedlings were grown in proprietary potting mixture in 45 mm pots under glasshouse conditions for 6 weeks and assessed for endophyte infection by immunoblotting (Simpson et al., 2012). Each CRISPR guide edit was successfully infected into at least 6 plant genotypes. Plants were allowed to mature for a minimum of a further 3 months under glasshouse conditions before being sampled for chemical analysis.

### Extraction of AR37 infected perennial ryegrass for chemical analysis

Pseudo-stems, the basal section of the tiller, were harvested from each endophyte-infected plant. Samples were harvested into liquid nitrogen and then transferred to a freeze drier (Freezone Plus12, Labconco Corporation, Kansas City, MI, USA). Once lyophilized the samples were ground and homogenised with a bead mill (Omni Bead Ruptor 24, Omni International Inc., Kennesaw, GA, USA) in a 7 mL vial using a ¼ inch zirconium bead (30 seconds at 4.5 m/s). Sub-samples (50 mg) were extracted with 1 mL of the prepared extraction solvent (80% v/v methanol with 0.54 ng/mL ergotamine, 0.202 ng/ml festuclavine, and 1.7 ng/mL homoperamine as internal standards) in 2 mL plastic vials for 1 hour by end-over-end rotation (30 Hz) in the dark. After centrifuging (5000 g, 5 min), the supernatant was transferred to 2 mL amber HPLC vials for analysis. Along with the samples, duplicate reference samples of AR37 (for quantifying the epoxyjanthitrems) were similarly extracted and analysed.

### LCMS analysis of indole diterpene compounds

Samples were analysed according to Berry et al. (2022). For the epoxyjanthitrems, due to the instability of epoxyjanthitrems as pure compounds, the peak areas and known concentrations of the reference samples (AR37) were used to determine response factors, which were used to subsequently quantify each epoxyjanthitrem compound in the samples. All values are an average of up to two guides, with each guide in at least 6 plant genotypes, per CRISPR gene edit (**Table 1**). **Table S2** shows the mass spectrometer parameters specific to each compound. For each indole diterpene a one-way ANOVA was used to compare the means of the inactivated genes to the AR37 wild type using MiniTab v19.1.1 (Minitab LLC, State College, PA, USA). A Fisher pairwise comparison was used to show significantly different means.

### LCMS analysis of epichloëcyclin compounds

Samples were analysed according to Johnson et al. (2015) with modification of the linear gradient profile (eluent A, aqueous 0.1% formic acid and eluent B, acetonitrile with 0.1% formic acid); time 0 mins (T_0_) at 5% B and held for 2 mins, T_10_ at 35% B, T_16_ at 95% B and held for 2 mins, followed by equilibration to initial conditions over the following 6 mins. Detection and quantitation were achieved using a LTQxl (Thermo Fisher Scientific, Waltham, MA, USA) in ESI positive ion mode collecting MS^1^ spectra in the range 300 – 1300 *m/z*. The raw data was interrogated using LCQuan v5.0 (Thermo Scientific Inc, San Jose, CA, USA). The epichloëcyclins specific to AR37 were detected as peaks of 562.3 and 570.3 *m/z* at 8.8 and 7.5 mins respectively. No attempt was made to quantify the epichloëcylins, as detection alone was sufficient to confirm infection with AR37.

### Bioinformatic analysis of off-target events

Genome for AR37 wild type and the CRISPR gene inactivated strains (multiple strains per guide) (**Table S3**) were assembled using MaSuRCA version 3.4.1 (Zimin et al., 2013) from reads previously generated by Illumina HiSeq and PacBio platforms. The trimmed MiSeq reads from each of the gene edited strains were aligned to the AR37 genome and the ANEp8-Cas9-LIC1 host plasmid sequence using the “mem” algorithm of BWA version 0.7.17-r118 (Li, 2013). The read alignments were then converted into the binary alignment (BAM) format using samtools version 1.8 (Li et al., 2009) for viewing in the Integrative Genomics Viewer (IGV) and for plasmid detection. Fuzznuc version 6.6.0.0 (EMBOSS) was used to find potential off-target sites in the AR37 genome by searching for each of the CRISPR target (protospacer and PAM) sequences with up to 5 mismatches in the first 21 bases. Up to 300 bases either side of the identified potential off target sites were visually inspected using IGV software. The view function of samtools version 1.8 (Li et al., 2009) was used to reveal any plasmid sequence integration into the genomes of the edits by counting the number of sequencing reads aligning to the plasmid (**Table S3**). Samples with more reads mapping to the plasmid than the control samples were confirmed visually using the IGV, whereas samples with less reads mapping to the plasmid than the control samples were considered ‘footprint-less’. Control DNA was generated by using an AR37 strain which had previously been genome sequenced twice, with and without spiking with plasmid, ANEp8-Cas9-LIC1, with the unspiked strain yielding no detectable vector contamination.

## Results

### AR37 and NEA12 epoxyjanthitrem cluster bioinformatics

**Figure 1:**
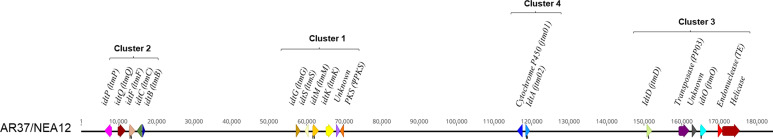
A comparison of syntenic analyses between AR37 and NEA12 epoxyjanthitrem biosynthetic clusters. Simplified gene location for epoxyjanthitrem, a group of indole diterpenes in *Epichloë* sp. *LpTG-3* strain AR37 and NEA12. Identification and comparison of the genetic region containing the gene clusters proposed to be involved in the production of epoxyjanthitrem compounds in AR37 and NEA12. Genes are depicted as coloured arrows with direction indicating the direction of the gene transcription, size proportional to the gene size, and colour indicating gene homology. Either the given name or function is labelled above the gene, with gene names in brackets referring to corresponding genes in Ludlow et al. (2019). Black lines under the arrows indicate the guide locations. The row of numbers are the base pairs across the region.

Given the structural similarities between janthitrems produced by *Penicillium janthinellum* (Nicholson et al., 2015) and epoxyjanthitrems produced by *Epichloë* sp. *Lp*TG-3 strain AR37 (Tapper and Lane, 2004), the nucleotide sequence of *P. janthinellum janD* (accession KF280651:44,433-45,819) was used as the query input sequence for blastn and blastx homology searches within *Epichloë* sp. *Lp*TG-3 strain AR37 genome assembly contigs. A hit was found by blastx with 52% protein level identity and 83% coverage. The corresponding gene was subsequently found within the same genetic scaffold containing known indole diterpene gene clusters by further blastn analyses using larger sequence regions surrounding the *janD* homologue following long-read DNA sequencing. In keeping with agreed naming conventions for indole diterpene genes of *Epichloë* sp., the *janD* equivalent gene was designated *idtD*. Coding sequences for the genes of this scaffold were determined using transcript information generated previously (Forester, unpublished) and the 19 proteins were subjected to blastp analysis for functional prediction. This led to the identification of four putative indole diterpene gene clusters containing 19 genes overall (**Figure 1**): cluster 1 and cluster 2 were homologous to lolitrem B cluster 1 and cluster 2 respectively (Young et al., 2006), cluster 3 containing the *janD* homolog (Nicholson et al., 2015), and cluster 4 containing genes of known indole diterpene biosynthesis. Another study recently reported on epoxyjanthitrems production in another *Epichloë* sp. *Lp*TG-3 strain, NEA12, highlighting four genes (*jtmO, jtmD, jtm01*, and *jtm02*) relevant to the biosynthetic pathway (Ludlow et al., 2019). Through genomic sequence comparison an identical genomic sequence to AR37, was shown for the indole diterpene gene cluster NEA12 (**Figure 1**). Genbank accession numbers for the *idt* gene clusters (and features therein) were assigned as ON500678 for AR37 and ON500679 for NEA12. A selected number of AR37 *idt* genes were then chosen for functional characterisation through CRISPR gene inactivation.

### Epoxyjanthitrem *idtM* CRISPR-Cas9 gene inactivation in AR37

**Figure 2:**
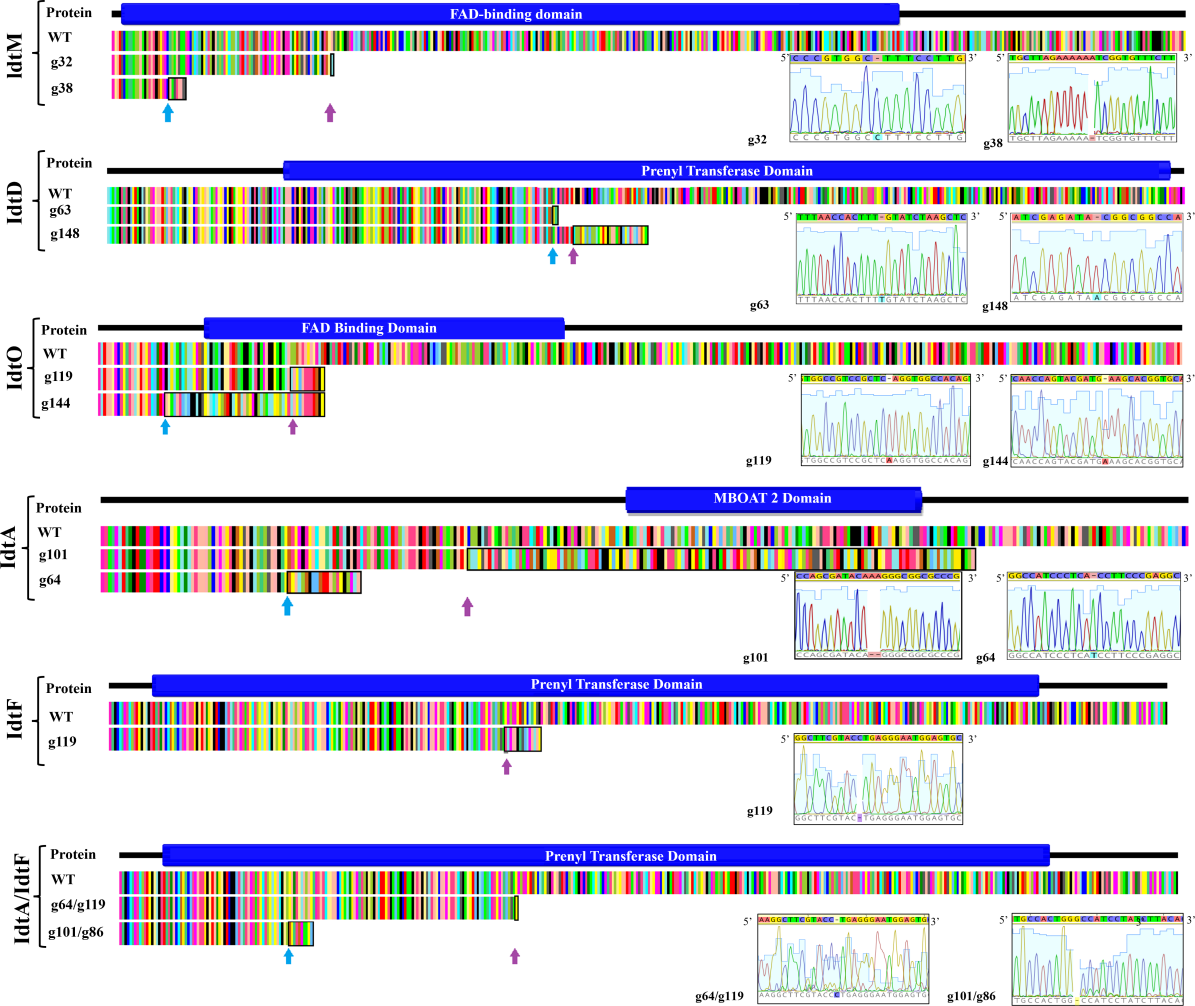
Resultant polypeptide and corresponding transcript of CRISPR gene edited indole diterpene genes in the proposed epoxyjanthitrem pathway. Pictorial depiction of the resultant polypeptide and corresponding transcript due to CRISPR gene inactivation of the indole diterpene genes idtM, idtD, idtO, idtA, idtF, and idtA/idtF when compared to the AR37 wild type transcript (WT) and the functional domain. The functional domain was predicted using the online software IntroPro Scan. The amino acid sequence of the AR37 wild type and CRISPR gene edited strains are colour coordinated by amino acid type with the indel site indicated in the CRISPR gene edited strains by a coloured arrow. The black box highlights the non-homologous amino acid sequence resulting from the indel. Description of the gene, guide, transformant number, indel, and frameshift is provided on the left-hand side of the figure and/or in the inserted table, with sequencing data provided in inserted chromatogram.

Expression of CRISPR-Cas9 through Cas9HygAMAccdB containing either guide 32 or guide 38 designed to target *idtM* in AR37, successfully resulted in 18% inactivation rate (from 16 transformants) or 68% inactivation rate (from 16 transformants) respectively (**Table 1**). One transformant from each guide was taken forward for further investigation; a single C insertion in transformant #12 for guide 32 or a single A deletion in transformant #8 for guide 38 (**Figure 2** and **Table 1**). *In planta* chemistry confirmed that both these *idtM* gene inactivated strains did not produce any indole diterpenes (**Table 2A**), with the endophyte presence confirmed through the detection of epichloëcyclins (Johnson et al., 2015), a characterised endophyte metabolite.

**Table 2:** Levels of indole diterpenes in AR37 wild type (WT) and AR37 strains with biosynthetic genes inactivated through CRISPR gene editing.

A		AR37 endophyte strain with CRISPR inactivated gene							
	Harvest 1	WT		*idtD*		*idtA*		*ltmM*	
	n	7		24		14		14	
	Paspaline	0.17	^B^	0.24	^A^	0.17	^B^	0	^C^
	Paspaline B	0.017	^B^	0.056	^A^	0.007	^BC^	0	^C^
	13-Desoxypaxilline	0.18	^B^	0.29	^A^	0.16	^B^	0	^C^
	Paxilline	0.04	^B^	0.16	^A^	0.02	^B^	0	^B^
	Terpendole E	0.05	^B^	0.08	^A^	0.05	^B^	0	^C^
	Terpendole I	0	^B^	0.332	^A^	0	^B^	0	^B^
	Terpendole C	0.005	^B^	0.26	^A^	0.003	^B^	0	^B^
	Terpendole K	0.015	^B^	3.307	^A^	0.012	^B^	0	^B^
	Terpendole N	0.008	^B^	4.429	^A^	0	^B^	0	^B^
	Epoxyjanthitriol	4.3	^B^	0	^C^	13.1	^A^	0	^C^
	Epoxyjanthitrem I	24.8	^A^	0	^B^	0.8	^B^	0	^B^
	Epoxyjanthitrem II	10.3	^A^	0	^C^	6.2	^B^	0	^C^
	Epoxyjanthitrem III	16.3	^A^	0	^B^	20.3	^A^	0	^B^
	Epoxyjanthitrem IV	9.8	^A^	0	^B^	0	^B^	0	^B^
**B**	
	**Harvest 2**	**WT**		***idtO***		***idtA***		***idtF***	
	n	4		21		22		22	
	Paspaline	0.08	^AB^	0.08	^B^	0.12	^A^	0.11	^A^
	Paspaline B	0.010	^A^	0.010	^A^	0.011	^A^	0.012	^A^
	13-Desoxypaxilline	0.77	^ABCD^	0.72	^B^	0.46	^D^	1.09	^A^
	Paxilline	0.02	^AB^	0.03	^AB^	0.03	^A^	0.02	^B^
	Terpendole E	0.03	^CD^	0.03	^D^	0.05	^A^	0.04	^BC^
	Terpendole I	0.012	^AB^	0.006	^B^	0.014	^A^	0.009	^B^
	Terpendole C	0.000	^AB^	0.000	^B^	0.000	^A^	0	^C^
	Terpendole K	0.002	^B^	0.002	^B^	0.003	^A^	0	^C^
	Terpendole N	0.004	^AB^	0.003	^B^	0.005	^A^	0	^C^
	Epoxyjanthitriol	2.6	^CD^	0	^D^	11.8	^B^	3.4	^C^
	Epoxyjanthitrem I	15.8	^B^	0	^C^	0.2	^C^	25.4	^A^
	Epoxyjanthitrem II	4.4	^A^	0	^B^	3.2	^A^	0	^B^
	Epoxyjanthitrem III	3.6	^B^	0	^C^	10.0	^A^	0	^C^
	Epoxyjanthitrem IV	2.7	^A^	0	^B^	0	^B^	0	^B^

### Epoxyjanthitrem *idtD* CRISPR-Cas9 gene inactivation in AR37

The same plasmid system was used to target *idtD* in AR37 through the expression of either guide 63 which resulted in a 62% inactivation rate (from 8 transformants) or guide 148 which resulted in a 100% inactivation rate (from 1 transformant) (**Table 1**). The two transformants taken forward where #113 which had a single T insertion and transformant #9 which had a single A insertion, respectively for each of the guides (**Figure 2** and **Table 1**). For both these *idtD* gene inactivated strains, *in planta* chemistry detected no epoxyjanthitrem compounds, but did detect paspaline, paspaline B, 13-desoxypaxilline, paxilline, and terpendole compounds E, I, C, N, and K at increased levels when compared to the AR37 wild type strain (**Table 2A**).

### Epoxyjanthitrem *idtO* CRISPR-Cas9 gene inactivation in AR37

When Cas9HygAMAccdB expressed guide 119 and guide 144, which both target *idtO* in AR37, a 10% inactivation rate (from 10 transformants) and 100% inactivation rate (from 7 transformants) was achieved for each guide, respectively (**Table 1**). Both transformants taken forward, #1 for guide 119 and #8 for guide 144, had an A insertion (**Figure 2** and **Table 1**). The *in planta* chemistry again detected no epoxyjanthitrem compounds. However, in contrast to the *idtD* inactivated strains, paspaline, paspaline B, 13-desoxypaxilline, paxilline, and terpendole compounds E, I, C, N, and K were detected at similar to that if the AR37 wild type strain (**Table 2B**).

### Epoxyjanthitrem *idtA* CRISPR-Cas9 gene inactivation in AR37

The *idtA* gene in AR37 was targeted through expression of guide 64 or guide 101 using the Cas9HygAMAccdB expression system and resulted in a 15% inactivation rate (from 20 transformants) or 33% inactivation rate (from 9 transformants) respectively (**Table 1**). Transformant #19-9 containing a single T insertion from guide 64 and transformant #3-4 containing a double A deletion from guide 101 were taken forward for further investigation (**Figure 2** and **Table 1**). For both these *idtA* gene inactivated strains, *in planta* chemistry showed that epoxyjanthitrems I and IV were eliminated or reduced, respectively, while the levels of epoxyjanthitrems II and III were equivalent and epoxyjanthitriol was increased, in comparison to the AR37 wild type strain. The detected levels of paspaline, paspaline B, 13-desoxypaxilline, paxilline, and terpendole compounds E, I, C, N, and K were comparable to those of the AR37 wild type strain (**Table 2A**).

### Epoxyjanthitrem *idtF* CRISPR-Cas9 gene inactivation in AR37

In AR37, *idtF* was targeted with the same plasmid system through the expression of guide 119 resulting in a 75% inactivation rate (from 4 transformants) (**Table 1**), with transformant #4 containing a C deletion taken forward in this study (**Figure 2** and **Table 1**). *In planta* chemistry detected, equivalent levels of epoxyjanthitriol and increased levels of epoxyjanthitrem I compared to AR37 wild type strain, while epoxyjanthitrems II, III, and IV were eliminated. The levels of paspaline, paspaline B, 13-desoxypaxilline, paxilline, and terpendole I were comparable to the AR37 wild type strain, while terpendoles C, K, and N were eliminated (**Table 2B**).

### Epoxyjanthitrem *idtA*/*idtF* CRISPR-Cas9 gene inactivation in AR37

Finally, double CRISPR gene inactivated strains were created using *idtA* inactivated protoplasts; IdtAg64 strain with *idtF* guide 119 or IdtA101 strain with *idtF* guide 86. The gene inactivation rates were 75% (from 4 transformants) and 33% (from 9 transformants) for the two *idtF* guides respectively (**Table 1**). The transformants taken forward were #4 containing a C insertion and transformant #8 containing a G deletion for *idtF* guides, respectively (**Figure 2** and **Table 1**). *In planta* chemistry showed levels of epoxyjanthitriol were increased while levels of epoxyjanthitrem I were heavily reduced. Again, epoxyjanthitrems II, III, and IV were eliminated along with terpendoles C, K, and N. Detected levels of paspaline, paspaline B, 13-desoxypaxilline, paxilline, and terpendole I were equivalent to the AR37 wild type strain (**Table 2B**).

### Target specific footprint-less CRISPR-Cas9 gene edits

Illumina genome sequencing was performed for all *idtM, idtD*, and *idtA* CRISPR gene edited strains as well as the AR37 wild type protoplast cell line, with genome sequencing of the remaining gene edited strains still currently underway for *idtO, idtF*, and *idtA/idtF*. Bioinformatic analysis of these sequenced genomes against a AR37 PacBio/Illumina consensus sequence concluded that fragments of the CRISPR plasmid (containing the guide, Cas9 gene, and hygromycin resistance gene) had not integrated into the genome for the selected transformants analysed (**Table S3**). Note that two reads mapped to vector sequence in whole genome reads from *idtM* strain transformed with guide 38, but both reads were subsequently ruled out as integrated vector sequence. In one case, this was because only one of the pair-end reads mapped to the vector, of which the alignment was to several vectors (not used in this study), whereas the other read aligned to sequence of bird origin, suggesting cross contamination during the sequencing process.

Although zero off-target sites were a criterion for protospacer design in the Geneious software, we additionally performed bioinformatic analysis on the same sequenced genomes to investigate possible off-target effects. To date, no off-target events have been detected using the search parameters employed, therefore we conclude that only the intended CRISPR gene edits are present.

## Discussion

### CRISPR-Cas9 gene inactivation in *Epichloë* sp. *Lp*TG-3 strain AR37

**Figure 3:**
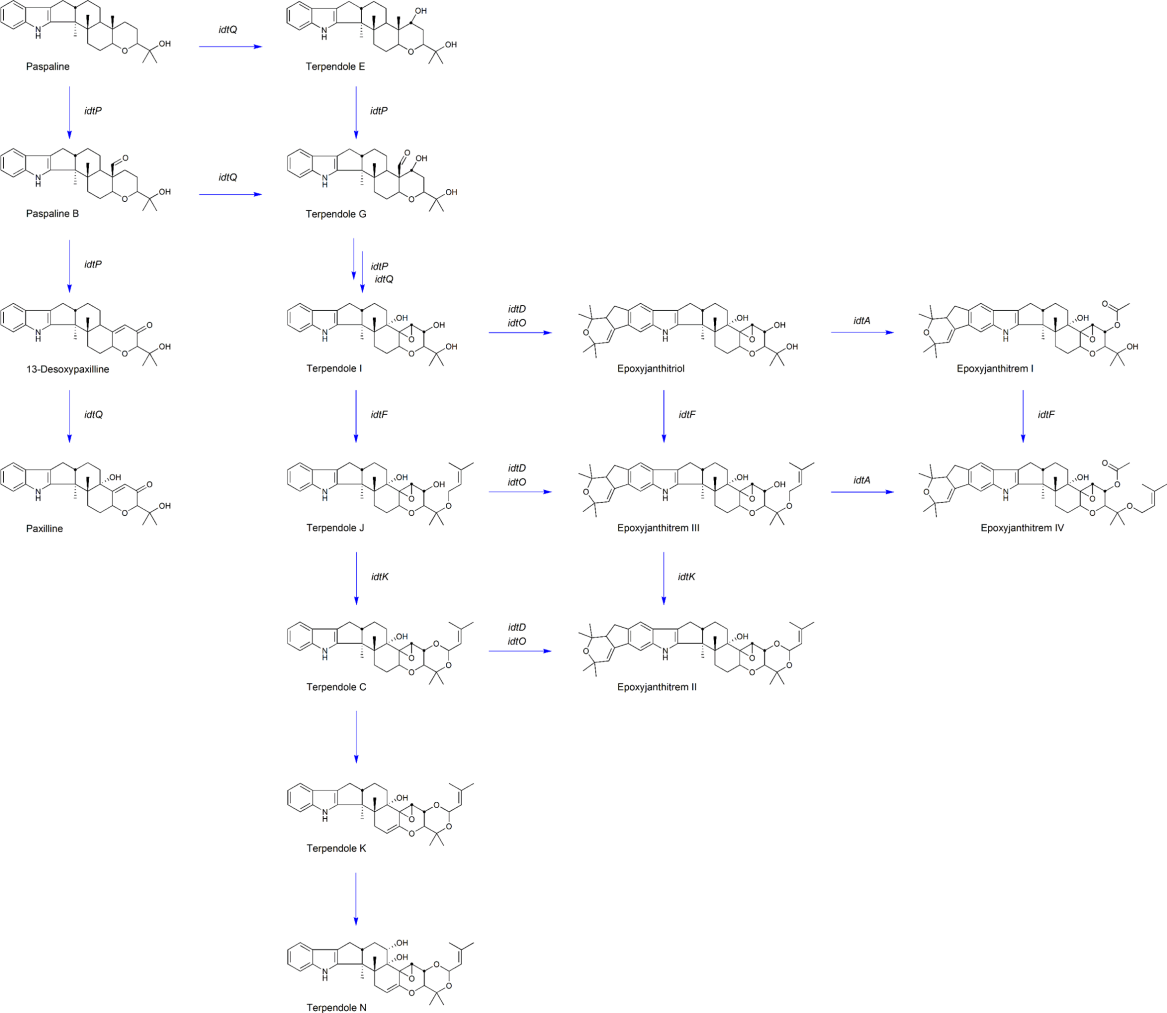
AR37 epoxyjanthitrem biosynthetic pathway. Key compounds proposed in the epoxyjanthitrem biosynthetic pathway in AR37. The compound name is labelled below the structure, with the corresponding gene involved in the compound production labelled next to the associated arrow.

For the first time, CRISPR-Cas9 has been successfully used in the commercial AR37 strain to validate the epoxyjanthitrem pathway. Through the gene inactivation of *idtM*, *idtD, idtO, idtA, idtF and idtA/F*, we have confirmed the role of each of these genes in epoxyjanthitrem biosynthesis as well as pathway intermediates at each step (**Figure 3**). In addition, this research has established that CRISPR-Cas9 can be used to carry out footprint-less gene inactivation to manipulate the secondary metabolite pathways of commercial *Epichloë* endophyte strains to provide greater chemical diversity, retaining or improving bioactivity against pests whilst eliminating animal toxicity.

### The role of *idtM* in epoxyjanthitrem biosynthesis

When *idtM*, one of the key enzymes involved in the first committed step in the epoxyjanthitrem pathway was inactivated, all indole diterpene compounds were eliminated (**Table 2A**). As *idtM* is part of the core indole diterpene gene set (Parker and Scott, 2004), this definitively links AR37 epoxyjanthitrem production to this pathway and fits the results from previous structural elucidation studies by Finch et al. (2020). Importantly, *idtM* gene inactivation eliminated all indole diterpenes, which are the only known bioactive compounds identified in AR37 to date.

### The role of *idtD* in epoxyjanthitrem biosynthesis

Ludlow et al. (2019) identified four clusters predicted to be involved in epoxyjanthitrem biosynthesis, and used RNAi to knock-down *idtD*, from cluster 3, resulting in a reduction of epoxyjanthitrem compounds. However, this current study conclusively links *idtD* to epoxyjanthitrem synthesis through an *idtD* CRISPR gene inactivation that removed all epoxyjanthitrem compounds (**Table 2A**). In addition, *idtD* encodes a protein which is predicted to be a prenyltransferase (Ludlow et al., 2019) and structural elucidation has confirmed that all five epoxyjanthitrem compounds contain a double prenylation on ring A (Finch et al., 2020). Overall, this supports the role of IdtD in the synthesis of all epoxyjanthitrem compounds *i.e.*, the initial gene involved in epoxyjanthitrem biosynthesis (**Figure 3**). Ludlow (2019) only investigated the levels of paxilline, which increased in the *idtD* knock-down and was also observed in this study (4-fold increase) (**Table 2A**). However, we additionally show that terpendole compounds are also produced by AR37, where they are barely detectable in the AR37 wild type, likely due to the rapid conversion of these compounds towards epoxyjanthitrems. The terpendoles are elevated to a greater extent (56-fold for terpendole C) when *idtD* is inactivated (**Table 2A**). The *idtD* gene inactivation therefore provides further confirmation and clarification of the terpendoles as intermediates of the epoxyjanthitrem biosynthesis pathway (**Figure 3**).

### The role of *idtO* in epoxyjanthitrem biosynthesis

Another gene identified in the same cluster as *idtD* by Ludlow et al. (2019)was *idtO*, predicted to encode a P450 monooxygenase (**Figure 1**). This was not an unexpected finding given that prenyltransferases and P450 monooxygenases are often associated together both in terms of the genome location as well as functionality in *Epichloëe.g.*, *idtF/idtK* and *idtE/idtJ* (Young et al., 2006; Young et al., 2009). When CRISPR was used to inactivate this gene, all epoxyjanthitrem compounds were removed (**Table 2B**) showing that IdtO, like IdtD, is also involved in the initial synthesis of all epoxyjanthitrem compounds *i.e.*, IdtD adds the two prenyl groups and IdtO cyclises these to form the ring structure. This is the first evidence presented that confirms *idtO* involvement in epoxyjanthitrem biosynthesis (**Figure 3**). However, the indole diterpene profile of the intermediate indole diterpene compounds upstream of the epoxyjanthitrem compounds for the *idtO* gene edit differs to the *idtD* gene edit (**Table 2A** and **Table 2B**). These results imply that the product of IdtD (predicted to be a diprenylated compound) is exerting a uncompetitive feedback inhibition where the product of one gene inhibits the upstream gene product stopping the terpendoles from accumulating (Guo et al., 2018) (**Figure 3**). Attempts thus far have failed to detect the predicted compound.

### The role of *idtA* in epoxyjanthitrem biosynthesis

In addition to *idtD* and *idtO* (located in cluster 3), *idtA* (located in cluster 4) (**Figure 1**) (Ludlow et al., 2019) was also inactivated using CRISPR. This resulted in the reduction of epoxyjanthitrem I, the removal of epoxyjanthitrem IV, the continued production of epoxyjanthitrems II and III, and the elevation of epoxyjanthitriol, in comparison to the AR37 wild type (**Table 2A**). This is the first evidence that confirms the involvement of cluster 4 in epoxyjanthitrem synthesis as well as the specific involvement of *idtA* in the direct synthesis of epoxyjanthitrem I and IV (**Figure 3**). It is hypothesised that the low levels of epoxyjanthitrem I may be due to promiscuous enzymatic activity of *Epichloë* and/or *Lolium* derived enzymes. This promiscuity is not routinely observed in other *Epichloë* indole diterpene pathways *e.g.*, the lolitrem B pathway, due to the much lower levels of indole diterpene compounds present in those pathways in relation to the epoxyjanthitrem pathway *i.e.*, promiscuous activity below levels of detection. Again, the predicted functionality of the protein encoded by *idtA* as an acetyltransferase (Ludlow et al., 2019) corresponds to the structures of the synthesised epoxyjanthitrem I and IV *e.g.*, the only epoxyjanthitrem compounds with an added acetyl group on carbon 43 (Finch et al., 2020). However, acetyltransferases are common in indole diterpene pathways in other species (Parker and Scott, 2004; Scott et al., 2013).

As seen in the *idtD* inactivated strains (**Table 2A**), removal of a gene involved in epoxyjanthitrem biosynthesis can result in changes to levels of upstream intermediate indole diterpene compounds. This would explain the changes in the amount of epoxyjanthitrems II and III as well as the accumulation of epoxyjanthitriol in the *idtA* inactivated strains even though *idtA* is not directly involved in synthesis of those compounds (**Table 2B**). By dramatically reducing the amount of epoxyjanthitrem I in the *idtA* inactivated strains this should reduce the tremorgenic effect of AR37, however there will be an associated reduction in insect bioactivity (Finch et al., 2020). The tremorgenic and bioactive properties of the remaining indole diterpene intermediate compounds would need to be investigated to get a complete understanding of the effect that changing the epoxyjanthitrem ratio would have on grazing livestock and invertebrate pests.

### The role of *idtF* in epoxyjanthitrem biosynthesis

Finally, *idtF* CRISPR inactivation eliminated epoxyjanthitrems II, III, and IV, while maintaining production of epoxyjanthitriol and epoxyjanthitrem I (**Table 2B**). IdtF is therefore involved in the epoxyjanthitrem pathway as well as the lolitrem B pathway, as previously reported, specifically in the direct synthesis of epoxyjanthitrems II, III, and IV in the epoxyjanthitrem pathway, which again corresponds to the function of IdtF as a prenyltransferase (Saikia et al., 2012; Schardl et al., 2013) (**Figure 3**).

In addition, when *idtF* is inactivated in an *idtA* inactivated strain, a hybrid indole diterpene profile is observed. In this case, epoxyjanthitriol was the major compound produced with a minimal amount of epoxyjanthitrem I, as observed in *idtA*, and the elimination of epoxyjanthitrems II, III, and IV as seen in *idtF* (**Table 2B**). This further confirms the roles of *idtA* and *idtF* as an acetyl transferase and prenyltransferase, respectively (**Figure 3**).

### Epoxyjanthitrem biosynthesis pathway discrepancies

Bioinformatic analysis of the epoxyjanthitrem gene cluster loci discovered in AR37 in this study and the epoxyjanthitrem gene cluster loci from Ludlow et al. (2019) re-analysed in this study, showed that the loci are identical within and between the clusters (**Figure 1**). In addition, both pathways predict an interconnected network of epoxyjanthitrem compounds, rather than a linear model, increasing compound diversity and therefore bioactivity, which is indicative of *Epichloë* indole diterpene pathways (Young et al., 2009; Ludlow et al., 2019) (**Figure 3**). In Ludlow et al. (2019), only paxilline and the four epoxyjanthitrems (I-IV) were detected and the proposed pathway was based on this limited data set. In contrast, the current study gathered data on a broader range of indole diterpene metabolites, including the terpendoles (**Table 2A** and **Table 2B**). The result of the removal of IdtD activity (namely the higher accumulation of terpendoles with low accumulation of paxilline) implies that the biosynthetic pathway progresses *via* the terpendoles, as seen in the biosynthetic pathway of lolitrem B, with terpendole I shown to be the first compound on which IdtD would act (Young et al., 2009). The biosynthetic pathway then simultaneously branches according to which enzyme interacts with successive products. Biosynthesis of epoxyjanthitrems *via* terpendole I also explains the presence of epoxyjanthitriol (Finch et al., 2020) that is absent in Ludlow’s (2019) pathway and which is analogous to lolitriol in the lolitrem B pathway (Schardl et al., 2013). Lastly, structural elucidation has confirmed that all epoxyjanthitrem compounds have α-stereochemistry at C-10, such as through the terpendoles, removing the need to invert the stereochemistry of the β-paxitriol precursor (Ludlow et al., 2019; Finch et al., 2020) (**Figure 3**).

### CRISPR-Cas9 plasmid delivery system in different fungal species

The CRISPR-Cas9 delivery system developed by Song et al. (2018) has now been successfully used in four different filamentous fungal species; *Aspergillus niger* (Song et al., 2018; Kun et al., 2021), *Venturia* (Rocafort et al., 2022), *Dothistroma septosporum* (McCarthy et al., 2022), and *Epichloë* sp. *Lp*TG-3 strain AR37 (this study) to inactivate a range of genes. These gene edits have been confirmed either through amplicon sequencing, high-resolution melting (HRM) curve, southern hybridisation, or genome sequencing. *Aspergillus* is a genetically tractable species and when this CRISPR-Cas9 system was developed and optimised for this specific species, the gene inactivation rate was highly efficient (75%-100%) although was dependent on the guide and gene targeted (Song et al., 2018; Kun et al., 2021). A similar gene inactivation rate was observed in the *Dothistroma* study (90%-100%), which was again gene and guide dependent (McCarthy et al., 2022). In contrast, *Venturia* is a genetically intractable species and when this CRISPR-Cas9 system (not developed or optimised for this specific species) was used, the gene inactivation rate dropped to 0%-16.7%, dependent on the guide. However this was the first time that targeted gene editing had been achieved in *Venturia* (Rocafort et al., 2022). In the current study, the gene inactivation rate of the genetically intractable *Epichloë* sp. *Lp*TG-3 AR37 strain fell within the *Aspergillus* and *Venturia* ranges of 15%-100%, depending on the guide and gene targeted. The difference in gene inactivation rate in the different species could be attributed to the guide used and gene targeted as well as the Cas9 gene being codon optimised to *Aspergillus* and/or the species natural non-homologous end joining (NHEJ) efficacy rate *i.e.*, high fidelity of NHEJ means lower mutation frequency. The *Aspergillus* studies, *Venturia* study, and *Dothistroma* study all used Geneious to select the guide, and also found that the guide genetic modification efficacy prediction did not correlate to the experimental rate (Song et al., 2018; Kun et al., 2021; Rocafort et al., 2022).

The advantage of using genome sequencing to confirm gene edits in this study is that it allowed for bioinformatic analysis of the whole genome to investigate the footprint-less and off-target nature of this CRISPR-Cas9 delivery system. For the first time, it has been confirmed that CRISPR can be implemented in *Epichloë* in a footprint-less manner to inactivate genes without off-target effects. This study highlights the importance of genome sequencing to identify gene edited strains for downstream applications. Furthermore, since a nucleic acid template was not used to guide Cas9 and the repair mechanism was through NHEJ, gene edits generated with this delivery system are classified as site-directed nuclease 1 (SDN-1), which are not regulated as genetically modified organisms (GMO) in selected jurisdictions (Lema, 2021; Kawall, 2021).

## Summary remarks

For the first time, the genetically intractable commercial AR37 strain of *Epichloë* sp. *Lp*TG-3 successfully underwent targeted genetic engineering using a CRISPR-Cas9 autonomously replicating system. The target genes were either previously associated with the *Epichloë* lolitrem B pathway (*idtM*, and *idtF*) or hypothesised to be associated with the epoxyjanthitrem pathway (*idtD*, *idtO*, and *idtA*) in AR37. The resultant CRISPR gene edits demonstrate that new biochemical variation can be created within the epoxyjanthitrem pathway with the accumulation of intermediate compounds and the removal of different epoxyjanthitrem compounds. The complete removal of the entire epoxyjanthitrem pathway through the precise gene inactivation of the *idtM* gene will be a useful tool for determining the potential for novel bioactivity of other secondary metabolite pathways that may be expressed by AR37. We have also confirmed the involvement of *idtD, idtO, idtA, and idtF* in the epoxyjanthitrem biosynthetic pathway based on the genetic dissection of these genes. This CRISPR-Cas9 system can now be applied to other *Epichloë* endophytes strains that remain genetically intractable to investigate genes of interest to advance *Epichloë* endophyte research. The manipulation of secondary metabolite pathways by CRISPR-Cas9 gene inactivation has the potential to deliver SDN-1 type endophytes for grasses with enhanced pest protection properties and reduced or no animal health and welfare issues.

## Data availability statement

The datasets presented in this study can be found in online repositories. The names of the repository/repositories and accession number(s) can be found below: https://www.ncbi.nlm.nih.gov/genbank/, ON500678, ON500679.

## Author contributions

RJ, LJ, WM, CV, DH, and JS designed the research. DH, JS, WM, NF, and PM performed experiments and analyzed data. TM, LJ, RJ, and WM wrote the manuscript. LJ, RJ, and CV acquired funding. All authors contributed to the article and approved the submitted version.

## Funding

Funding was provided through the Ministry of Business, Innovation and Employment (MBIE) Partnerships Investment Contract C10X1902 and with commercial partners Grasslanz Technology Ltd and PGG Wrightson Seeds Ltd under contract CV/IP/274.

## Acknowledgments

Concordia University for providing the CRISR-Cas9 plasmid delivery system ANEp8_Cas9_LIC plasmid and ANEp8_Cas9 plasmid (Song et al., 2018). Sarah Finch, and Pranav Chettri for reviewing this manuscript.

## Conflict of interest

The authors declare that this study received funding from Grasslanz Technology Ltd and PGG Wrightson Seeds Ltd. The funders were not involved in the study design, collection, analysis, interpretation of dat or the writing of this article. They were however involved in the decision to submit it for publication.

## Publisher’s note

All claims expressed in this article are solely those of the authors and do not necessarily represent those of their affiliated organizations, or those of the publisher, the editors and the reviewers. Any product that may be evaluated in this article, or claim that may be made by its manufacturer, is not guaranteed or endorsed by the publisher.
