# Supplementary figures and images for: Dissection of the epoxyjanthitrem pathway in Epichloë sp. LpTG-3 strain AR37 by CRISPR gene editing
Source: Front Fungal Biol. 2022 Aug 10;3:944234. doi: 10.3389/ffunb.2022.944234 (PMC10512260; doi:10.3389/ffunb.2022.944234)

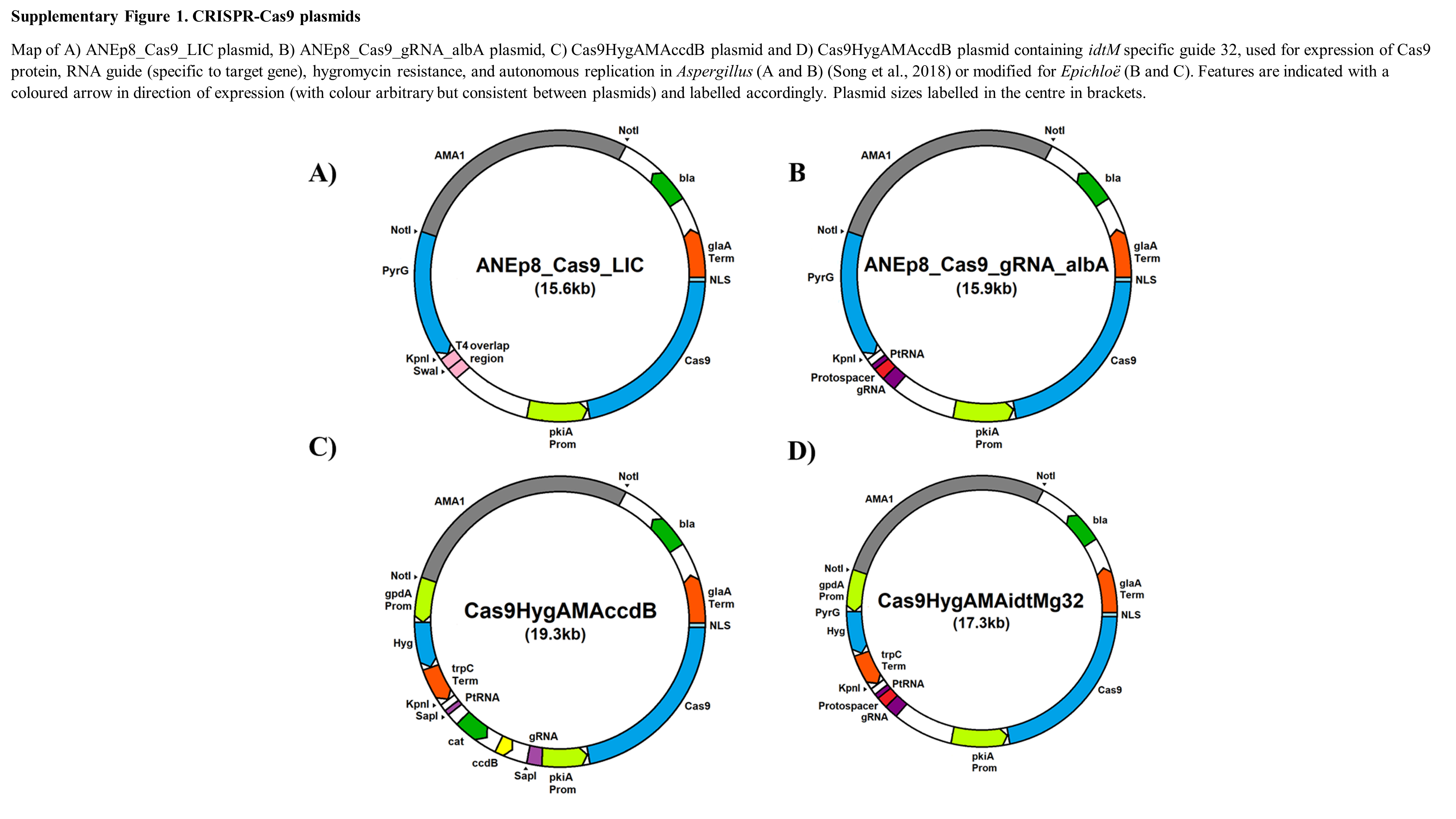

Supplement: Supplementary file 4 [file Image_1.tif]
